# Elevated biomarkers of neural injury in older adults following head-down bed rest: links to cardio-postural deconditioning with spaceflight and aging

**DOI:** 10.3389/fnhum.2023.1208273

**Published:** 2023-09-26

**Authors:** Andrew P. Blaber, Farshid Sadeghian, Donya Naz Divsalar, Isobel A. Scarisbrick

**Affiliations:** ^1^Department of Biomedical Physiology and Kinesiology, Simon Fraser University, Burnaby, BC, Canada; ^2^Department of Physical Medicine and Rehabilitation, Center for Regenerative Biotherapeutics, Mayo Clinic, Rochester, MN, United States

**Keywords:** neural injury markers, bed rest immobilization, spaceflight analog, baroreflex, electromyogram (EMG), orthostatic intolerance (OI), cardio-postural control

## Abstract

**Introduction:**

Prolonged physical inactivity with bed rest or spaceflight is associated with cardiovascular and neuromuscular deconditioning; however, its impact on neural integrity of cardio-postural reflexes and possible mitigation with exercise has not been examined. We assessed the association between the physiological deconditioning of bed rest immobilization with neural injury markers and the effects of 60–75 min of daily exercise.

**Methods:**

Data were collected as part of a randomized clinical trial (clinicaltrials.gov identifier: NCT04964999) at the McGill University Medical Centre. Twenty-two 55- to 65-year-old healthy volunteers gave informed consent and took part. Within sex, participants were randomly assigned to exercise (60- to 75-min daily) or control (inactive) groups and spent 14 days in continuous 6° head-down tilt. Neural injury [neurofilament light chain (NfL), glial fibrillary acidic protein (GFAP), total tau (t-Tau), myelin basic protein (MBP), brain-derived neurotrophic factor (BDNF), ubiquitin carboxy-terminal hydrolase L1 (UCH-L1)], as well as interleukin-6 (IL-6), tumor necrosis factor alpha (TNF-α), and insulin-like growth factor 1 (IGF-1) biomarkers were measured before, during, and after bed rest. The false discovery rate with Huber M-estimation was used to correlate changes in biomarkers with cardiovascular and muscular function changes over bed rest.

**Results:**

Bed rest elevated NfL, GFAP, TNF-α, and IL-6 in all participants and reduced IGF-1 in females only. With standing, changes in heart rate, blood pressure, and lower limb muscle motoneuron activity correlated with changes in TNF-α and BDNF. Baroreflex control, leg muscle maximal voluntary contraction, and postural sway are correlated with GFAP and NfL. Exercise participants had fewer interactions than control participants, but significant correlations still existed, with both groups exhibiting similar reductions in orthostatic tolerance.

**Discussion:**

An hour of daily exercise in older persons otherwise immobilized for 2 weeks did not abate bed rest-induced increases in serum signatures of neural injury or pro-inflammatory markers. Exercise reduced the number of physiological interactions of biomarkers, but significant cardio-postural correlations remained with no protection against post-bed rest orthostatic intolerance. The identification of associations of inflammatory and neural injury biomarkers with changes in cardio-postural physiology and exercise points to biotherapeutic opportunities and improved exercise interventions for astronauts and individuals in bed rest.

**Clinical trial registration:**

https://www.clinicaltrials.gov/search?cond=NCT04964999, identifier: NCT04964999.

## Introduction

Spaceflight produces observable physiological changes in humans (Vernikos and Schneider, 2010), leading to time-dependent adaptation processes. Weightlessness instantaneously removes physical processes that depend on weight for their effect and is experienced by bodily systems in different ways. Eventually, weightlessness affects every part of the body, either directly or indirectly.

Neural activity driven by motor and sensory experience plays essential roles in neural development, plasticity, and aging (Stimpson et al., 2018). Data from 60 days of head-down tilt bed rest, an analog for spaceflight, revealed neurological deficits in the baroreflex. On the first day of recovery, the formerly healthy younger individuals had a 61% reduction in total muscle-pump baroreflex (reflex changes in skeletal muscle activation to changes in blood pressure) gain and only a partial return 8 days later (Xu et al., 2020). The cardiac baroreflex (reflex changes in heart rate to changes in blood pressure) was also reduced in these same participants immediately after bed rest, but it recovered 8 days later (Blaber et al., 2022).

Causal analysis of the cardiac and muscle-pump baroreflex feedback loops following bed rest revealed a significant reduction in the coupling along the neural reflex segment of the bi-directional baroreflex interaction (Xu et al., 2020; Blaber et al., 2022) (blood pressure driving heart rate and muscle pump) but not for the mechanical coupling of heart rate and muscle pump on blood pressure. These data suggested an impairment or degradation of the neural regulation of blood pressure independent of cardiac or skeletal muscle mechanics. However, it could not be determined from these data where, in the reflex signal integration or efferent pathway, the deficit occurred.

From these results, we hypothesized that inactivity induced by bed rest immobilization and the fluid shift accompanying 6° head-down tilt affects the central nervous system (CNS) and activates neurodegenerative processes. Since exercise training has been shown to improve myelin outcomes, including related elevations in spinal cord IGF-1 (insulin-like growth factor 1) in adult mice (Yoon et al., 2016), we hypothesized that an exercise program involving the major upper and lower body muscles would reduce signs of neural insult occurring with bed rest.

Such an exercise program was proposed and supported by the Canadian Space Agency (CSA), the Canadian Institutes of Health Research (CIHR), and the Canadian Frailty Network (CFN) as part of the Canadian Aging and Inactivity Study (CAIS) involving older adults placed in 14 days of 6° head-down tilt bed rest (HDBR). HDBR is often used as an analog of spaceflight because it induces inactivity, unloads the musculoskeletal system in the head-to-feet axis (*z*-axis), and simulates the fluid shift seen in spaceflight. Our team was one of eight chosen to participate in this first-of-its-kind project that combined older adults in a space analog with a space-based exercise intervention (Hedge et al., 2022).

Although our foundational research was based on young to middle-aged volunteers (Xu et al., 2020), given the increased age of the CAIS participants, we expected and observed similar declines in cardiac and muscle-pump baroreflex over 14 days (Sadeghian et al., 2022). Moreover, we found the exercise program was not effective in preventing reductions in cardiac baroreflex and only partially blunted the decline in muscle-pump baroreflex. Based on these results, we concluded that the exercises did not preserve orthostatic reflexes. This was reflected in reduced orthostatic tolerance in our 5-min stand test after bed rest, where 7 of 20 participants, equally distributed between control and exercise, could not complete the test after bed rest when all were successful during baseline (Sadeghian et al., 2022). None were able to complete a standard 15-min 80° head-up tilt test immediately after bed rest (Hajj-Boutros et al., 2023).

Mobility, especially ambulatory mobility, is essential for quality of life and independence. With aging, ambulatory ability tends to deteriorate due to sarcopenia (aging-associated degenerative loss of skeletal muscle quality, mass, and strength; Phillips, 2015) and dynapenia (aging-associated loss of muscle strength not arising due to muscular and/or neurological diseases; Clarke and Manini, 2008). Avoiding the cycle of bed rest-induced muscle wasting and further reduced ambulation requires immediate intervention after hospital admission to remobilize the patient as early as possible (Singh et al., 2008; Goswami, 2017). There is evidence to show that strength training can evoke muscle hypertrophy and positive changes in neuromuscular function, even at >80 years of age (Aagaard et al., 2010).

The goal of this research was to use the unique opportunity of 6° head-down tilt bed rest to further our understanding of cardiac and muscle-pump baroreflexes in relation to the development of orthostatic intolerance in older adults. In this pilot study, we examined inflammatory and neurodegenerative markers to further assess their relationship with neural deficits in cardiac and muscle-pump baroreflexes and with physical and physiological declines commonly associated with bed rest. We also explored the impact of 60–75 min of daily exercise to counter the bed rest-induced cardiovascular and skeletal muscle deconditioning.

## Materials and methods

### Study design and testing protocols

Our research was conducted as part of a clinical trial implemented through collaboration with the Canadian Institutes of Health Research (CIHR), the Canadian Frailty Network (CFN), and the Canadian Space Agency (CSA). The overall aim was to investigate the effects of bed rest inactivity/immobilization on older persons (55–65 years old), with specific emphasis on exercise interventions and applications to spaceflight. The Center for Innovative Medicine (CIM) of the McGill University Health Centre Research Institute (RI-MUHC) conducted the trial, which comprised four 26-day bed rest campaigns during which 5–6 participants per campaign were placed in HDBR as a ground-based analog to spaceflight.

Recruitment provided a balance between biological sex across test groups, with half of the participants receiving bed rest-specific exercise counter measures (CMs) (Hedge et al., 2022), while the other half served as controls, receiving stretch and joint movement physiotherapy. The exercises consisted of three sessions each day of high-intensity interval training (HIIT), low-intensity aerobic activity, and lower body strength exercises, resulting in an average of 1 h of daily physical activity (Hedge et al., 2022). Workouts were performed in the head-down tilt position using specially modified equipment. The intensity of the exercise CMs was adjusted to the individual participants' maximal performance and tolerance prior to bed rest. All other standards of care were consistent between the control and exercise groups (Hedge et al., 2022; Sadeghian et al., 2022).

Ethical approval for the research was obtained from the research ethics board of the MHUC. Research and data collection associated with our study were approved by the Office of Research Ethics at Simon Fraser University. The research was conducted in compliance with the guidelines and regulations of the above agencies. The inclusion and exclusion criteria were previously reported (Hedge et al., 2022; Sadeghian et al., 2022). In brief, older persons (55–65 years old) and healthy male and female individuals, with women being menopausal, were included. Volunteers were included if they spent at least 2.5 h of moderate- to vigorous-intensity aerobic activity per week. Sedentary people and people who were addicted to exercise were excluded. Out of 219 healthy volunteers, 22 entered the study after passing screening and giving informed consent. The participants signed written informed consent and agreed to be available at MHUC for the entire 26-day study period.

### Data collection

#### Blood sampling and analysis

We quantified well-studied serum biomarkers of neural injury across the neuronal and glial substrates of the central nervous system (CNS). Specifically, we quantified changes in the well-studied marker of astrocyte reactivity, glial fibrillary acidic protein (GFAP) (Stukas et al., 2023). In addition, we quantified both tau and neurofilament light chain (NfL), which have emerged as sensitive serum markers of CNS neurodegeneration (Kuhle et al., 2015; Dage et al., 2016; Mattsson et al., 2017; Thompson et al., 2018). We also quantified serum levels of markers that could arise from the CNS or peripheral organs, including brain-derived neurotrophic factor (BDNF) and ubiquitin C-terminal hydrolase L1 (UCH-L1). BDNF and UCH-L1 are associated with neuron and glial development and survival and have been linked to neurodegenerative conditions and aging in prior studies (Ziegenhorn et al., 2007; Reinicke et al., 2019). Insulin-like growth factor 1 (IGF-1) and two inflammatory cytokines, tumor necrosis factor alpha (TNF-α) and interleukin-6 (IL-6), all of which have the potential to impact CNS integrity, were also quantified.

**Figure 1:**
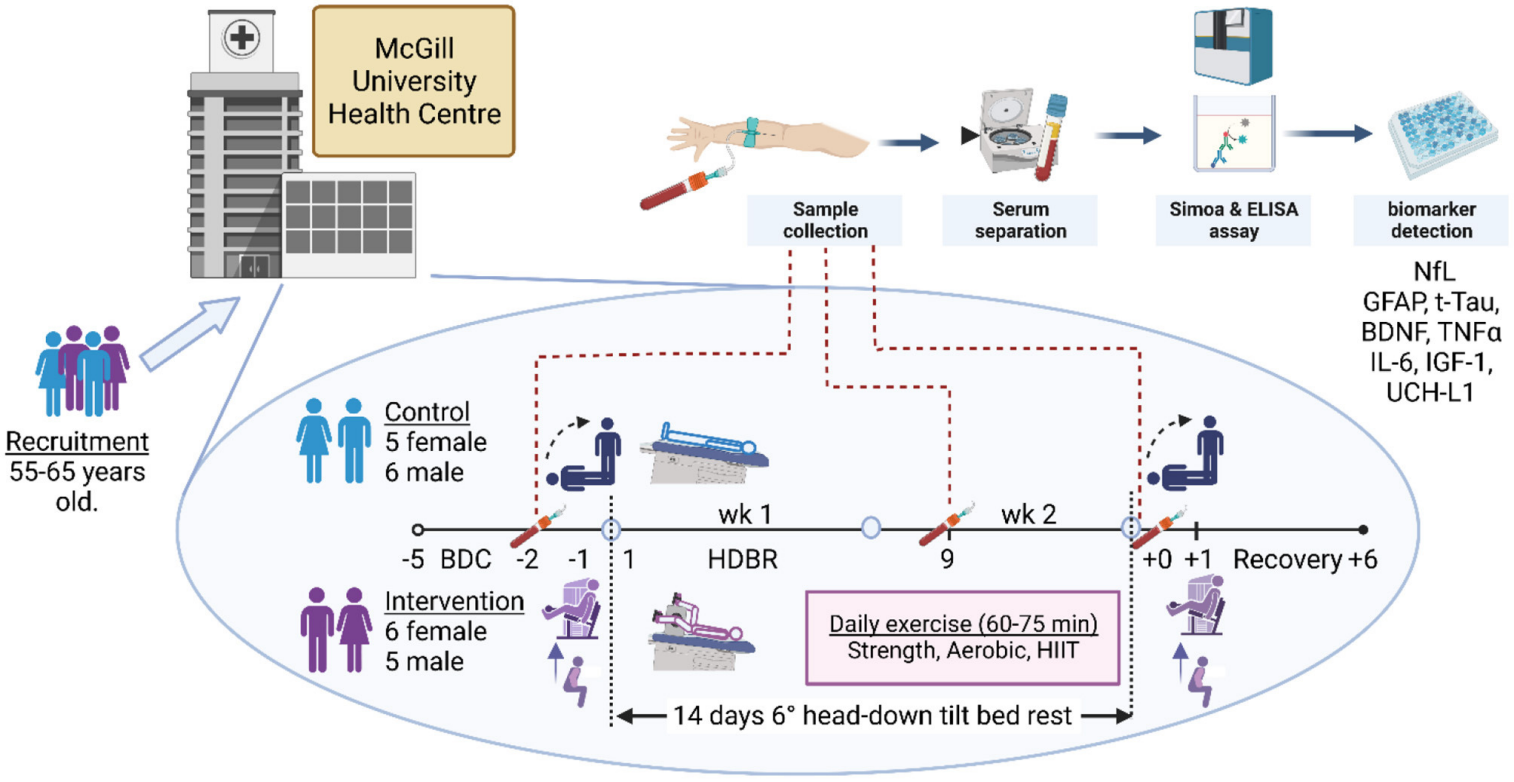
Staff screened volunteers between the ages of 55 and 65 years old from the Montreal region at the Centre for Innovative Medicine at the McGill University Health Centre. Following recruitment and screening, 22 participants, equally represented by males and females, consented to take part and provided blood samples on all days of collection. Within each sex, participants were randomly assigned to either the control or the exercise intervention. The daily exercise routine comprised upper and lower body strength regimens and aerobic and high-intensity interval training dispersed through three sessions a day. Daily exercise time ranged from 60 to 75 min. In the first 5 days, the participants were ambulatory for baseline data collection (BDC-5 to BDC-1), following which they entered the 14-day head-down bed rest (HDBR) phase of the study (HDBR1 to HDBR14). Immediately after HDBR, the participants returned to ambulation recovery (R+0 to R+6) within the facility, exiting on R+7. Blood draws were taken in the mornings of BDC-2, HDBR9, and R+0 after overnight fasting and prior to eating. Thereafter, blood was centrifuged, and blood sera and plasma were frozen in liquid nitrogen and stored at −80°C. Hemoglobin was analyzed at MUHC, while NfL (neurofilament light chain), GFAP (glial fibrillary acidic protein), TNF-α (tumor necrosis factor alpha), IL-6 (interleukin-6), t-Tau (total tau protein), IGF-1 (insulin-like growth factor 1), BDNF (brain-derived neurotrophic factor), and UCH-L1 (ubiquitin carboxy-terminal hydrolase L1) were analyzed by independent laboratories at the University of British Columbia and Simon Fraser University. The supine-to-stand baroreflex and postural sway test were performed on the mornings of BDC-1, the last day before HDBR, and R+0. The physical jump, muscle activity, and strength tests were conducted on BDC-1 and R+1. Created with Biorender.com.

Standard venipuncture from the antecubital vein of the arm was performed during baseline data collection 2 days before bed rest (BDC-2), the ninth day of bed rest (HDBR9), and the day they came out of bed rest (R+0) (Figure 1). One allotment was transported to the MUHC laboratory for hemoglobin analysis. Another was centrifuged, and sera were frozen in liquid nitrogen and stored at −80°C before being shipped overnight on dry ice to the Wellington Laboratory at the Djavad Mowafaghian Center for Brain Health at the University of British Columbia. Single Molecular Array (Simoa^®^, Quanterix Corp., USA) HD-kit analysis was performed for NfL, GFAP, UCH-L1, IL-6, TNF-α, and t-Tau. IGF-1, myelin basic protein (MBP), and BDNF were analyzed from aliquots of the same serum samples with multiplex Luminex ELISA (Thermo Fisher Scientific) in the Brockman and Brumme Laboratory at Simon Fraser University.

#### Variables from supine-to-stand test

The cardio-postural control system was activated and evaluated using a supine-to-stand (StS) test (Blaber et al., 2009; Garg et al., 2013, 2014; Rodriguez et al., 2017; Verma et al., 2017; Xu et al., 2017) prior to bed rest (BDC-1, Figure 1) and twice after recovery (Sadeghian et al., 2022). For comparison with serum analysis, we used the StS results on BDC-1 and R+0 that were closest to the blood draws (BDC-2, R+0; Figure 1). The StS test, previously described (Sadeghian et al., 2022), was conducted in the morning, 1 h after the Canadian Space Agency (CSA) standard 15-min tilt test. Participants were instrumented in the supine position for electrocardiogram (IX-BIO4, iWorx, USA), non-invasive beat-to-beat blood pressure (Portapres, FMS Technologies, the Netherlands), and bi-lateral electromyography (EMG) (8-channel Bagnoli, Delsys Inc., Natick, MA, USA) of the soleus (S), medial gastrocnemius (MG), lateral gastrocnemius (LG), and tibialis anterior (TA). To determine the placement locations for the EMG sensors, the recommendations of the SENIAM project (Hermens et al., 1999) were followed. Data were collected at a rate of 1,000 Hz with National Instruments USB-6218 16-bit data capture equipment and LabVIEW 2013 software from National Instruments Inc. (Austin, TX, USA).

Once the participant was instrumented, the lights were turned off, and they closed their eyes for the 5-min baseline data collection. Afterward, the participant was assisted into a standing position. One researcher swept their legs off the bed, and another assisted with raising their torso. Each participant stood with their feet parallel and 5 cm apart, without moving their feet, and with their arms relaxed at their sides. Their eyes remained closed with an imaginary gaze at eye level for the subsequent 6 min of quiet stance.

#### Jump test

A countermovement jump was used to measure whole-body power. The jump maneuver started with an upright stance with hands on the waist. Participants quickly squatted down and pressed firmly on a force plate to jump as high as possible with hands on hips to prevent arm movement. Participants performed mild aerobics and three sets of warm-up squats before the test. Each participant recorded three maximum jump efforts with their heads held as high as possible throughout the test. A rest period of 60–90 s between each jump was allowed for recovery. The vertical ground reaction force was used to determine the subject's acceleration, velocity, power profile, flight altitude, and total time in the air. Body mass was assessed by having the subjects stand motionless for 30 s on a force plate. All participants were at least 1.5 h post-prandial and had not performed any vigorous exercise in the previous 18 h.

#### Muscle characteristics

To measure the strength of various leg muscles, a multi-joint muscle dynamometer (Biodex Systems, USA) was used. The device was used to examine both isometric and isotonic maximum voluntary contractions (MVC). Isometric contractions involve holding a muscle contraction without joint movement, while isotonic contractions involve moving a joint through a specific range of motion while maintaining a constant force output. Simultaneous EMGs from the S, MG, and LG were collected using the same procedure described for supine-to-stand. During the isometric test, the participants sat and pushed against the device using their ankle muscles (S, LG, and MG), aiming to produce the strongest possible isometric contractions. The dynamometer was attached to the feet to measure the strength and power of the ankle plantar flexor muscles. During the isotonic assessments, each participant moved their ankle joint back and forth while applying force against the device, which generated a resistance load. The test consisted of three repetitions of either isometric or isotonic movements, and the MVC for each muscle was determined based on the highest muscle contraction recorded across all three tests. Throughout the examination, participants received both verbal and visual feedback. Prior to the test, the subjects received instruction on appropriate exercise techniques and equipment orientation.

The soleus, medial gastrocnemius, and lateral gastrocnemius muscles make up most of the plantar flexor muscles of the ankle joint (Sadeghian et al., 2018, 2019). During isometric contractions, the magnitude of the force produced by these muscles was recorded with the Biodex. The non-linear and dynamic aspects of the ankle joint can be disregarded since isometric contractions minimize the effects of parameters related to joint angle (Leardini et al., 2000; Sadeghian et al., 2019). Therefore, the S, MG, and LG muscles were responsible for producing the measured force, and therefore the maximum force produced by each muscle may be calculated as a ratio of its peak EMG signals (Troiano et al., 2008).

#### Statistical analyses

Statistical analyses were performed using JMP 16 (SAS Institute).

##### Serum biomarkers

Data are presented as mean (95% lower and upper confidence limits). Data within groups (sex, intervention) were tested for normality using the Shapiro–Wilk goodness-of-fit test. If the variables were normally distributed, a repeated measures ANOVA was used to assess time-dependent changes of the biomarkers as a function of sex (female, male) and intervention (control, exercise). If the RM-ANOVA revealed significant differences, *post-hoc* tests were performed using Tukey's HSD. If the data were determined to be not normally distributed, a Friedman test was used.

##### Correlations between serum biomarkers and physiological measures

To investigate the possible relationship between changes in participant physiological characteristics and the serum biomarkers, data were converted from absolute pre- and post-values to the change (Δ) from pre- to post-HDBR. Negative values represented a decrease in biomarker or physiological value, and positive values represented an increase after bed rest.

Given the small number of participants in the subject groups and the possible non-linear relationship between biomarkers and physiological measurements, Spearman's rank correlation was used to assess the strength of correlations between changes in biomarker concentration and changes in physical assessments made by the participants before and after bed rest. Data are presented using the correlation coefficient (ρ).

With multiple comparisons, the false discovery rate (FDR) (Benjamini and Hochberg, 1995) was used to estimate the significance of the correlations. The response screening function with the robust Huber M-estimation method was used (Huber and Ronchetti, 2009). The threshold was set to positive false discovery rate (pFDR) < 0.05 (Korthauer et al., 2019). Data are quoted as variable (pFDR).

To assess the interaction of biomarkers and physical measurements with changes observed in the cardiac and muscle-pump baroreflexes, we performed a stepwise multiple regression analysis. Variables entered or exited the model to provide a minimum Bayesian information criterion (BIC). We also performed discriminant analysis on our data to investigate whether biomarkers and physiological variables could be used to differentiate between the categorical groups of sex (male, female) and intervention (control, exercise), as well as the four subgroups (male control, male exercise, female control, female exercise). A stepwise procedure was used to enter and exit variables until there was no overlap between the groups.

## Results

One participant dropped out of the study on day 3 of HDBR, leaving 22 participants equally divided between exercise [five males 58.0 (0.8) years, six females 60.6 (0.1)] and control [six males 60.8 (1.5), five females 56.6 (0.5); mean (SEM)]. Control and exercise participants were not significantly different in aerobic fitness as measured by ${\dot{\text{V}}\text{O}}_{2}$peak (mean ± SD; control: 30.2 ± 5.8; exercise: 32.0 ± 6.6 mL·min^−1^·kg^−1^). Two participants withdrew from the study on R+2 for medical reasons following the completion of the post-bed rest physical measurements; however, three participants (two males and one female) were missing muscle and/or baroreflex data because of poor signal quality. Therefore, correlational analysis of the biomarkers with physical measurements contains the results from 19 participants.

### Blood analysis

Aliquots for serum analysis were taken from the same sample used for the blood biomarkers. Hemoglobin was used as a systemic control protein for all analyses using an identical statistical approach. No significant change in hemoglobin was found over the time course of BDC-2 through R+0 (*p* = 0.854) with an overall value of 135 (132, 139) g/L. Male participants had consistently higher hemoglobin values than female participants [146 (141, 150) g/L, 126 (121, 130) g/L, respectively].

### Serum assays

**Figure 2:**
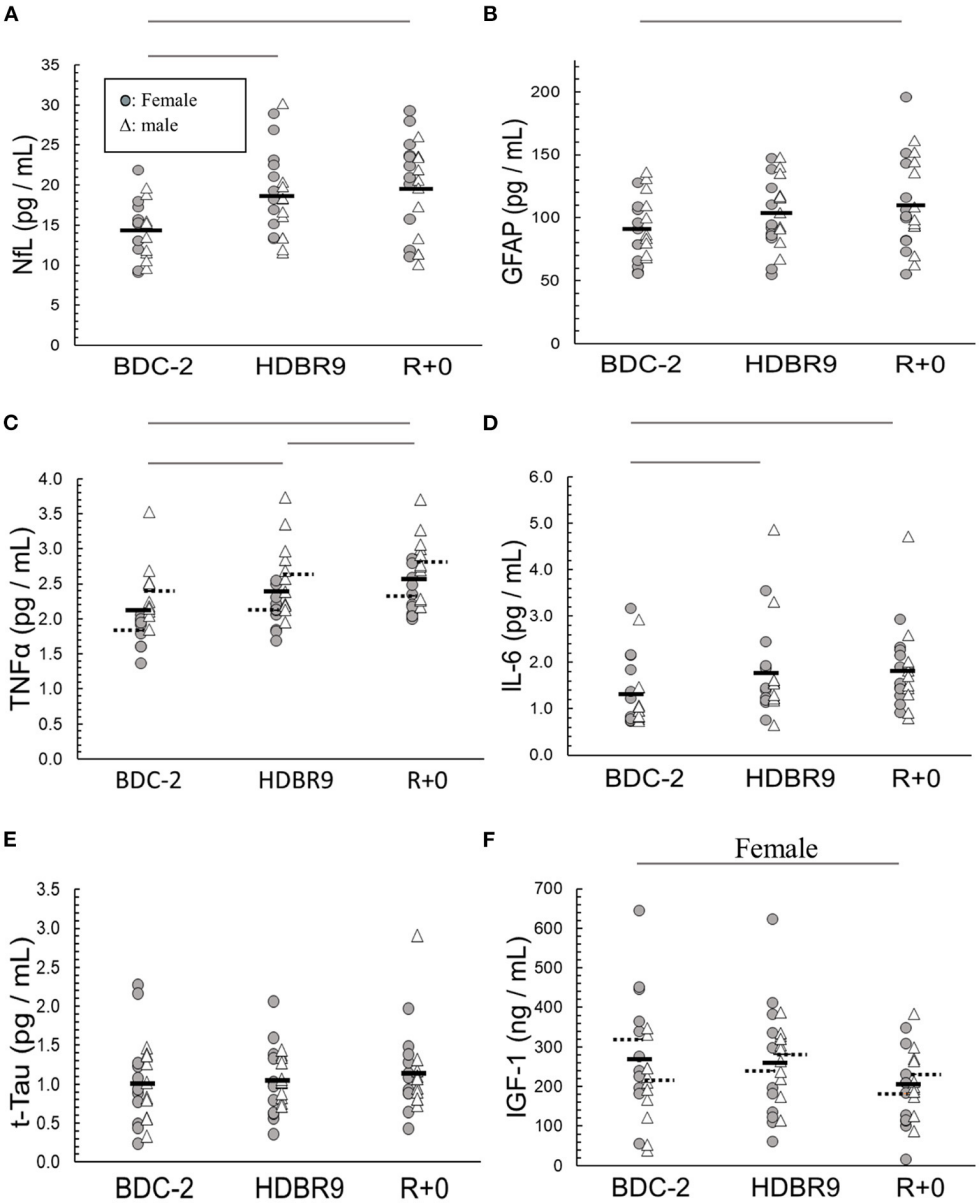
Main effect changes over time in **(A)** NfL (neurofilament light chain), **(B)** GFAP (glial fibrillary acidic protein), **(C)** TNF-α (tumor necrosis factor alpha), **(D)** IL-6 (interleukin-6), **(E)** t-Tau (total tau), and **(F)** IGF-1 (insulin-like growth factor 1) from baseline data collection 2 days before (BDC-2) head-down tilt bed rest (HDBR) through 9 days of HDBR (HDBR9) to the first day of recovery (R+0). Females: circles (O); males: triangles (Δ). Within the data, a dashed horizontal line represents the mean of male and female data: left solid line (female mean); right solid line (male mean). The horizontal lines above the figures represent significant differences (*p* < 0.05) between days on each end of the line. For I, significance was with female participants only.

We analyzed sera to investigate whether bed rest, intervention, or sex significantly altered the concentrations of our selected biomarkers. All (*p* ≥ 0.05) but UCH-L1 (*p* = 0.027) were found to be normally distributed (Shapiro–Wilk) within sex and intervention. NfL increased from the beginning to the end of bed rest (*p* < 0.0001), mostly by day 9 (Figure 2A; Table 1), with no sex (*p* = 0.34) or intervention (*p* = 0.80) effects. GFAP increased from the beginning to the end of bed rest (*p* < 0.0001) (Figure 2B; Table 1) with no sex (*p* = 0.53) or intervention (*p* = 0.56) effects. Bed rest elevated TNF-α (*p* = 0.006) (Figure 2C; Table 1), increasing HDBR9 and R+0 with consistently higher values in males [2.61 (0.12) pg/mL] than females [2.01 (0.12) pg/mL] (*p* = 0.006). IL-6 increased significantly from the start of bed rest to HDBR9, when it plateaued (Figure 2D, *p* = 0.0006) (Table 1) with no effect of sex (*p* = 0.97) or intervention (*p* = 0.90). No group effects were observed for t-Tau [1.06 (0.35) pg/mL, *p* = 0.41] (Figure 2E; Table 1). IGF-1 decreased significantly in females from pre- to post-bed rest but not in males (regardless of intervention group, *p* = 0.018) (Figure 2F). No consistent group changes in BDNF or UCH-L1 were observed from the beginning to the end of bed rest (*p* = 0.87, *p* = 0.42, respectively) with intervention (*p* = 0.18, *p* = 0.78, respectively) or by sex (*p* = 0.11, *p* = 0.26, respectively). MBP was not detected with the assay used (below the detection limit in sera; assay range: 0.2–15 ng/mL).

**Table 1:** Data from serum samples taken during baseline 2 days before head-down tilt bed rest (BDC-2), on the ninth day of bed rest (HDBR9), and on the day of remobilization (R+0).

		**Pre-bed rest (BDC-2)**		**6°head-down tilt bed rest (HDBR 9)**		**Post-bed rest (R**+**0)**	
**Protein**	**Sex**	**Control**	**Exercise**	**Control**	**Exercise**	**Control**	**Exercise**
NfL (pg/mL)	Male Female	15.3 (10.9, 19.6) 15.0 (10.2, 19.7)	12.9 (8.2, 17.6) 14.1 (9.8, 18.4)	18.2 (13.9, 22.6) 18.4 (13.7, 23.2)	16.2 (11.4, 20.9) 21.1 (16.7, 25.4)	18.2 (13.9, 22.5) 21.1 (16.7, 25.8)	17.9 (13.1, 22.6) 20.9 (16.6, 25.2)
GFAP (pg/mL)	Male Female	105 (78, 131) 87 (58, 116)	89 (60, 118) 81 (55, 108)	116 (89, 142) 95 (66, 124)	103 (74, 132) 100 (74, 127)	116 (90, 143) 111 (82, 140)	103 (74, 132) 108 (81, 134)
TNF-α (pg/mL)	Male Female	2.47 (2.12, 2.81) 1.92 (1.54, 2.30)	2.30 (1.92, 2.68) 1.76 (1.41, 2.11)	2.68 (2.33, 3.02) 2.22 (1.84, 2.59)	2.59 (2.21, 2.97) 2.04 (1.70, 2.39)	2.81 (2.46, 3.15) 2.42 (2.04, 2.79)	2.80 (2.42, 3.18) 2.24 (1.90, 2.59)
IL-6 (pg/mL)	Male Female	1.32 (0.61, 2.04) 1.23 (0.44, 2.02)	1.02 (0.23, 1.81) 1.58 (0.86, 2.30)	2.25 (1.53, 3.00) 1.28 (0.49, 2.06)	1.32 (0.53, 2.11) 2.06 (1.35, 2.78)	2.02 (1.30, 2.74) 1.40 (0.62, 2.19)	1.73 (0.94, 2.52) 2.04 (1.32, 2.76)
t-Tau (pg/mL)	Male Female	1.07 (0.65,1.49) 0.96 (0.50, 1.41)	0.79 (0.34, 1.25) 1.56 (0.74, 1.58)	0.97 (0.55, 1.38) 1.02, (0.56, 1.48)	1.19 (0.73, 1.65) 1.03 (0.61, 1.45)	1.00 (0.58, 1.42) 1.13 (0.56, 1.48)	1.34 (0.88, 1.80) 1.06 (0.64, 1.48)
IGF-1 (ng/mL)	Male Female	218 (119, 318) 293 (184, 402)	173 (65, 282) 324 (225, 424)	251 (151, 350) 171 (63, 280)	294 (185, 403) 332 (232, 431)	239 (139, 337) 187 (78, 296)	193 (85, 302) 170 (70, 268)
BDNF (ng/mL)	Male Female	12.8 (9.9, 14.9) 12.9 (10.2, 15.7)	12.6 (9.8, 15.3) 9.0 (6.5, 11.5)	12.4 (9.9, 14.9) 11.9 (9.2, 14.7)	12.4 (9.7, 15.2) 9.2 (6.7, 11.7)	13.5 (11.0, 16.0) 11.6 (8.8, 14.4)	12.4 (9.6, 15.2) 9.9 (7.4, 12.5)
UCH-L1 (pg/mL)	Male Female	3.9 (0, 10.5) 5.2 (0, 11.0)	1.2 (0, 8.7) 6.8 (0.3, 13.2)	2.7 (0, 9.7) 5.4 (0, 12.7)	3.3 (0, 10.4) 9.6 (3.1, 16.1)	3.2 (0, 9.8) 3.9 (0, 11.0)	1.8 (0, 8.8) 7.2 (0.7, 13.6)

Mean (95% lower and upper confidence limits) for: NfL, neurofilament light chain; GFAP, glial fibrillary acidic protein; TNF-α, tumor necrosis factor alpha; IL-6, interleukin-6; t-Tau, total tau protein; IGF-1, insulin-like growth factor 1; BDNF, brain-derived neurotrophic factor; UCH-L1, ubiquitin carboxy-terminal hydrolase L1.

Biomarker sensitivity. *Quanterix HD-1/HDX:* NfL (LoD: 0.104 pg/mL; LoQ: 0.241 pg/mL; dynamic range: 0–2,000 pg/mL), GFAP (LoD: 0.221 pg/mL; LoQ: 0.467 pg/mL; dynamic range: 0–4,000 pg/mL), TNF-α (LoD: 0.016 pg/mL; LoQ: 0.034 pg/mL; dynamic range: 0–400 pg/mL), IL-6 (LoD: 0.0055 pg/mL; LoQ: 0.01 pg/mL; dynamic range: 0–120 pg/mL), t-Tau (LoD: 0.019 pg/mL; LoQ: 0.061 pg/mL; dynamic range: 0–360 pg/mL), UCH-L1 (LoD: 1.74 pg/mL; LoQ: 5.45 pg/mL; dynamic range: 0–40,000 pg/mL). *Luminex:* IGF-1 (assay range: 2.9–3,000 ng/mL), BDNF (assay range: 0.4–400 ng/mL).

### Changes in physical/physiological measurements with bed rest

#### All participants

**Figure 3:**
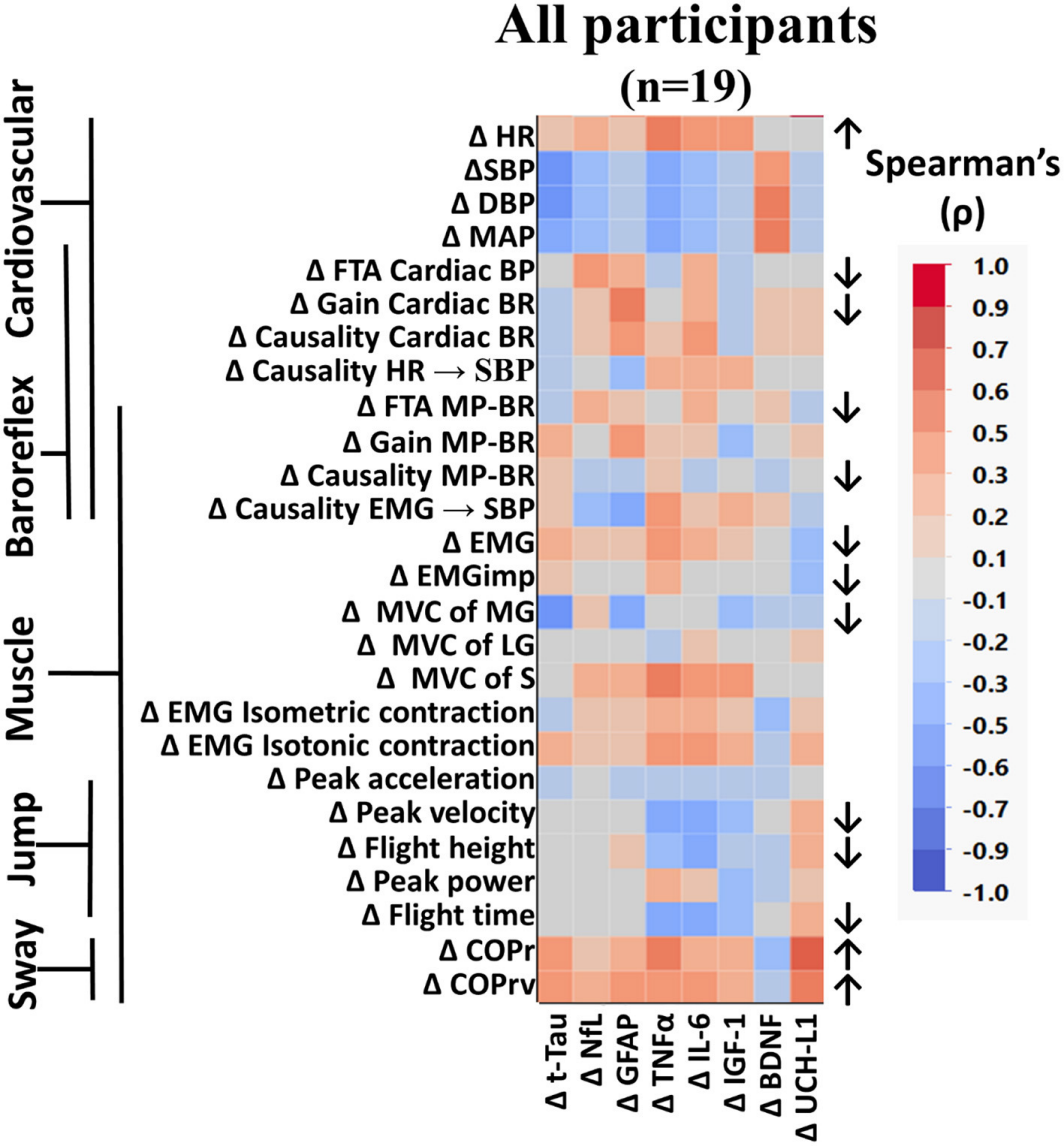
Color heat map of Spearman's rank correlation analysis for all participants. Changes in serum biomarkers (abscissa) from baseline (R−0 to BDC-2) were compared to changes in physical and physiological function (ordinate) from baseline (R−0 to BDC-1). Significant correlations are listed in Table 2. All data were found to be normally distributed using the Shapiro–Wilk test. Arrows to the right of the heat map indicate a significant increase (↑) or decrease (↓) in physical/physiological variables from BDC-2 to R+0 (mean significantly different from 0). Student's *t*-test was performed for normally distributed data. Non-normally distributed data were tested using the Wilcoxon signed rank test. HR, heart rate; SBP, systolic blood pressure; DBP, diastolic blood pressure; MAP, mean arterial pressure; FTA, fraction time active; MP-BR, muscle-pump baroreflex; MVC, maximum voluntary contraction; MG, medial gastrocnemius; LG, lateral gastrocnemius; S, soleus; EMG, electromyography; COPr, average center of pressure sway radius; COPrv, average COP sway velocity. Biomarkers: Tau, Tau protein; NfL, neurofilament light chain; GFAP, glial fibrillary acidic protein; TNF-α, tumor necrosis factor alpha; IL-6, interleukin-6; BDNF, brain-derived neurotrophic factor; IGF-1, insulin-like growth factor 1; UCH-L1, ubiquitin carboxy-terminal hydrolase L1.

When all participants were grouped together, several measured variables were found to be significantly altered from baseline to the end of bed rest (Figure 3). Similar to what we previously reported from these data (Hedge et al., 2022; Sadeghian et al., 2022), heart rate (HR) was increased, cardiac and muscle-pump baroreflexes were reduced, and muscle activation during standing was lower following bed rest. In these analyses, we observed reduced cardiac FTA and gain along with reduced muscle-pump FTA and causality. This was accompanied by reductions in summed muscle EMG and EMG impulse during standing.

**Table 2:** Changes in physiological measurements at the end of bed rest (R+0, R+1) compared to before bed rest (BDC-1, BDC-2) for the four different conditions.

**Measure**	**Female**		**Male**	
	**Control (*****n*** = **4)**	**Exercise (*****n*** = **6)**	**Control (*****n*** = **4)**	**Exercise (*****n***= **5)**
ΔHR (bpm)	25 (5, 44)^*^	20 (10, 30)^*^	14 (3, 25)^*^	20 (4, 35)^*^
ΔSBP (mmHg)	−20 (−48, 8)	−4 (−40, 32)	27 (−29, 83)	−20 (−46, 7)
ΔDBP (mmHg)	−4 (−10, 2)	9.7 (−14, 34)	16 (−2, 33)	1 (−6, 8)
ΔMAP (mmHg)	−9 (−17, 0)^*^	4.7 (−23, 32)	18 (−8, 44)	−3.7 (−14, 7)
ΔFTA cardiac BR	−28 (−87, 31)	−6.8 (−24, 11)	−1.7 (−17, 13.5)	−18 (−43, 7)
ΔGain cardiac BR (ms/mmHg)	−2.6 (−4.5, −0.8)	−1.4 (−2.4, −0.4)^*^	−1.8 (−3.2, −0.3)^*^	−4 (−6.7, −1.3)^*^
ΔCausality cardiac BR	0.05 (−0.01, 0.1)	0.00 (−0.2, 0.2)	−0.01 (−0.09, 0.05)	−0.04 (−0.1, 0.02)
ΔCausality HR → SBP	−0.03 (−0.1, 0.04)	0.04 (−0.07, 0.1)	0.01 (−0.07, 0.08)	0.01 (−0.25, 0.09)
ΔFTA MP–BR	−10 (−24, 4)	−8.7 (−16, −1)^*^	−7.5 (−40, 25)	−23 (−46, 0)
ΔGain MP-BR (μV/mmHg)	0.05 (−0.6, 0.7)	−0.1 (−0.3, 0.6)	−0.26 (−0.42, −0.1)^*^	−0.4 (−1, 0.2)
Δcausality MP-BR	−0.07 (−0.16, 0.01)	−0.03 (−0.25, 0.19)	−0.1 (−0.2, −0.01)	−0.06 (−0.17, 0.04)
Δcausality EMG → SBP	−0.03 (−0.2, 0.15)	−0.01 (−0.08, 0.09)	0.01 (−0.02, 0.01)^*^	0.00 (−0.07, 0.07)
ΔEMG (μV)	−12.5 (−38.6, 13.6)	−18.7 (−58, 20.9)	−88 (−260, 84)^*^	−7.3 (−45, 30)
ΔEMGimp (μV·s)	−24 (−40, −9)^*^	−24 (−49, 1)^*^	−96 (−268, 76)^*^	−16 (−49, 17)
ΔMVC of MG (N)	0 (−40, 40)	−10 (−30, −1)^*^	−10 (−30, 100)	−3 (−17, 11)
ΔMVC of LG (N)	0 (−50, 50)	−25 (−55, 5)^*^	20 (−10, 50)	5 (−30, 40)
ΔMVC of S (N)	15 (−90, 110)	−3 (−15, 80)	−6 (−20, 6)	−2 (−30, 20)
ΔEMG isometric (μV)	6 (−90, 100)	−35 (−60, −6)^*^	0 (−60, 60)	0 (−75, 75)
ΔEMG isotonic (μV)	35 (−16, 23)	−145 (−244, −45)^*^	0 (−120, 120)	−15 (−110, 80)
Δpeak acceleration (m·s^−2^)	0.2 (−0.3, 7.5)	0.06 (−0.2, 0.3)	−0.3 (−1.1, 0.5)	−0.32 (−0.7, 1.3)
Δpeak velocity (m·s^−1^)	−0.2 (−0.26, −0.15)	−0.14 (−0.26, −0.01)^*^	−0.1 (−0.3, 0.15)	−0.2 (−0.5, 0.06)^*^
Δpeak height (m)	−3.8 (−4.6, −3.1)^*^	−2.3 (−4.5, −0.3)^*^	−2.17 (−6.4, 2)	−3.43 (−7, 0.15)^*^
Δpeak power (W)	0.5 (−0.5, 1.4)	−0.7 (−1.6, 0.2)^*^	−0.75 (−1.9, 0.4)	−0.02 (−0.57, 0.51)
Δflight time (s)	−0.04 (−0.05, −0.03)^*^	−0.03 (−0.05, −0.002)^*^	−0.02 (−0.07, 0.03)	−0.04 (−0.1, −0.01)^*^
ΔCOPr (mm)	6.8 (−5.4, 19)	3.8 (0.08, 7.5)^*^	1 (−0.8, 2.8)	4.95 (1.4, 8.4)^*^
ΔCOPrv (mm·s^−1^)	27 (−17, 71)	9.8 (3.7, 16)^*^	11.7 (−3.9, 27)	7.8 (−3, 18.5)

Values are the mean (95% lower and upper confidence limits).

*Standing portion of the supine-to-stand test:* HR, heart rate; SBP, systolic blood pressure; DBP, diastolic blood pressure; MAP, mean arterial pressure; Cardiac BR, cardiac baroreflex; FTA, fraction time active; EMG, average rectified electromyogram signal; EMGimp, EMG impulse—EMG signal summed over a heartbeat; MP-BR, muscle-pump baroreflex. *Supine measurements:* MVC, maximum voluntary contraction; MG, medial gastrocnemius; LG, lateral gastrocnemius; S, soleus; EMG isometric, maximum EMG signal during maximum force against a fixed object; EMG isotonic, average EMG produced with maximum force exertion to move an object at a fixed velocity. *Jump test:* Peak acceleration, maximum acceleration during the ascending phase; peak velocity, maximum upward velocity; Peak height, maximum vertical displacement of the center of mass; Peak power, maximum mechanical power generated in the push phase; flight time, time during which an individual is airborne. *Postural sway:* COPr, average center of pressure sway radius; COPrv, average COP sway velocity.

* Indicates the mean is significantly different from zero (*p* < 0.05, Wilcoxon signed rank test), with expected increases for HR, COPr, and COPrv and decreases in all other variables.

Here we report for the first time, in older persons, the effects of HDBR on changes in postural sway, maximal voluntary contraction of calf muscles, calf muscle EMG during maximal isometric and isotonic contraction, and the maximal jump test (Figure 3). There was a significant reduction in force production from the MG, not the LG or S. No changes were seen in EMG output during isometric or isotonic contractions. The ability to jump was reduced with lower peak velocity, height, and flight time. Postural sway during the stand test was more pronounced with increases in radius (COPr) and velocity (COPrv).

#### Sex and intervention

**Figure 4:**
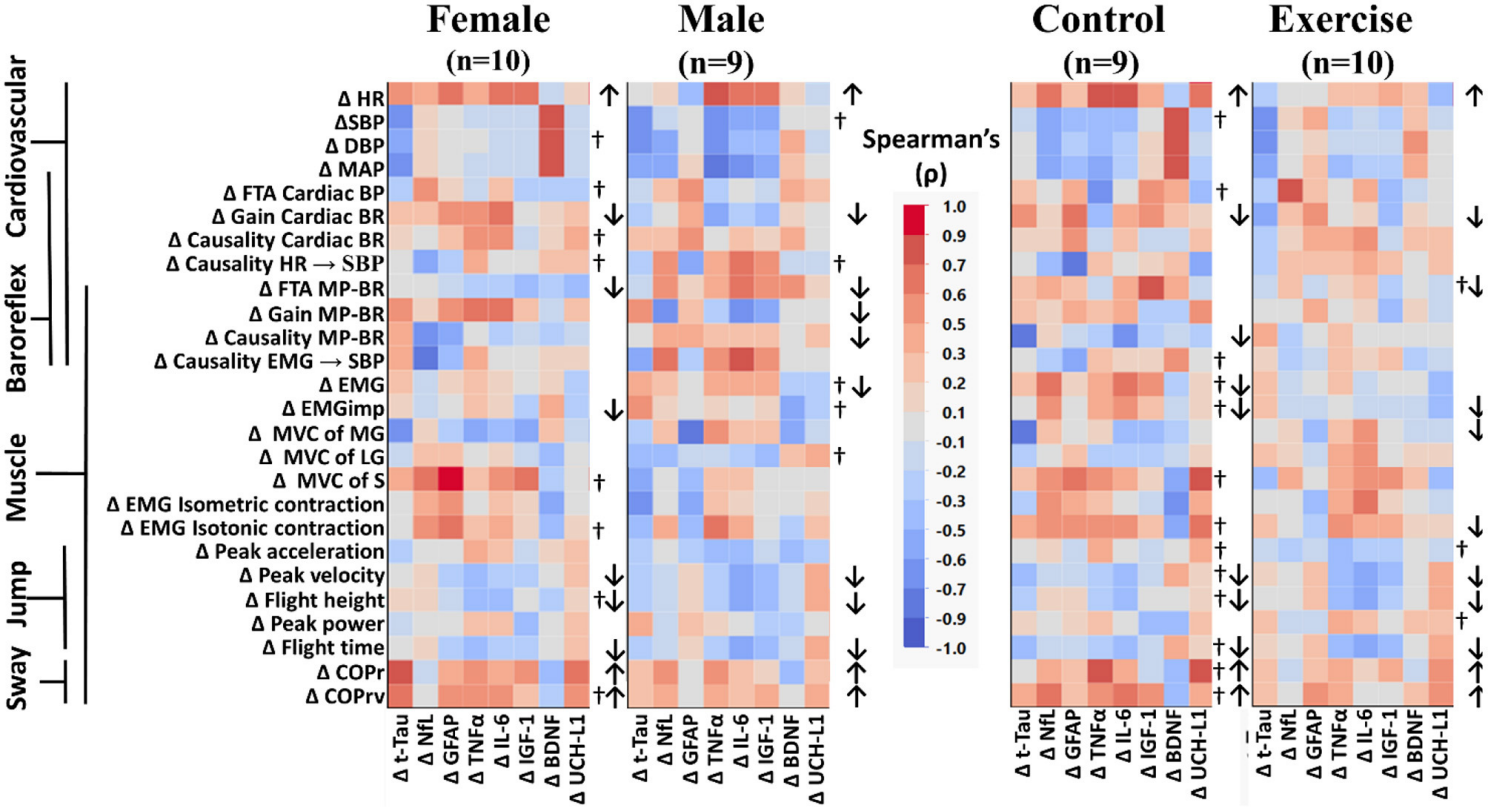
Color heat maps of Spearman's rank correlation analysis by sex (female, male) and by intervention (control, exercise). Changes in serum biomarkers (abscissa) from baseline (R−0 to BDC-2) were compared to changes in physical and physiological function (ordinate) from baseline (R−0 to BDC-1). Significant correlations using a robust false discovery rate are listed in Table 3. Physiological data were tested for normal distribution with the Shapiro–Wilk test. Data that were found to be not normally distributed are marked (†) on the right of their respective sex or intervention row. Arrows to the right of the heat map indicate a significant increase (↑) or decrease (↓) in physical/physiological variables from BDC-2 to R+0 (mean significantly different from 0). Student's *t*-test was performed for normally distributed data. Non-normally distributed data were tested using the Wilcoxon signed rank test. HR, heart rate; SBP, systolic blood pressure; DBP, diastolic blood pressure; MAP, mean arterial pressure; FTA, fraction time active; MP-BR, muscle-pump baroreflex; MVC, maximum voluntary contraction; MG, medial gastrocnemius; LG, lateral gastrocnemius; S, soleus; EMG, electromyography; COPr, average center of pressure sway radius; COPrv, average COP sway velocity. Biomarkers: Tau, Tau protein; NfL, neurofilament light chain; GFAP, glial fibrillary acidic protein; TNF-α, tumor necrosis factor alpha; IL-6, interleukin-6; BDNF, brain-derived neurotrophic factor; IGF-1, insulin-like growth factor 1; UCH-L1, ubiquitin carboxy-terminal hydrolase L1.

When compared across sex (Figure 4, left) or across intervention (Figure 4, right), similar results to the analysis of all participants were found, with small variances in the component variables in a category. The increase in HR with reduced cardiac baroreflex gain, along with reduced jump characteristics (peak velocity, flight height, and flight time), and increased postural sway (COPr and COPrv) were consistent in all groups (Figure 4).

When comparing across sex, there were significant declines in more muscle-pump indices for the male participants (FTA, gain, causality) compared to the females (FTA). In female participants, there was a decline in total EMG output, while in males, the decline was observed with EMGimp. Control participants had reduced muscle-pump causality, while the exercise group had reduced FTA. There was an observed decrease in both total muscle EMG and EMGimp in the control group, with the exercise group only showing a decrease in EMGimp. Finally, only the exercise group had changes in EMG output during isotonic contractions or muscle MVC. The exercise group had lower MVC in the medial gastrocnemius and lower EMG during isotonic contractions (Figure 4).

### Correlations of changes in biomarker levels with physical/physiological changes in bed rest

We used Spearman's rank correlation analysis to determine possible relationships between an individual's change in a biomarker and changes in their main cardiovascular and muscular system physiological measurements (Figure 2) for all participants and across the different participant groups (Figure 3). In the overall Spearman's analysis (Figure 3), we observed a wide range of strengths in positive and negative relationships between changes in physiological measurements and the biomarkers. In the group analysis, correlations revealed distinct patterns of biomarker interactions with changes in physiological measures from bed rest for sex (Figure 4, left) and for intervention (Figure 4, right). Female and male participants showed similar numbers of darker shaded (higher correlation) compartments; whereas, compared to controls, exercise participants had a much lighter pattern, indicative of less and weaker correlations. Some of the physiological variables measured were not normally distributed (†) with double the incidence in females compared to males. There were three non-normally distributed variables in the exercise group; however, the control group had more than four times that number.

A quantitative assessment of the correlations using the false discovery rate with M-Huber estimation (*p* ≤ 0.05) revealed some trends in biomarker distribution between the cardiovascular and muscular systems across all participants (Table 3).

**Table 3:** Correlation across all participants of individual changes in physical/physiological system markers from baseline (R0 to BDC-2) with the change in serum biomarkers (R1 to BDC4) using false discovery rate analysis with robust Huber M-estimation (*p* < 0.05).

**System**	**Unit**	**Δ Physical/physiological variable**	**Participants (*n* = 20) Δ biomarker**
Cardiovascular	Cardiac	HR	TNF-α (0.029)
	Vascular	Systolic BP	TNF-α (< 0.014), BDNF (0.039)
		Diastolic BP	BDNF (0.022)
		Mean BP	BDNF (0.022)
	Cardiac BR	FTA	NfL (0.036)
		Gain	
		Causality	
	HR → SBP	Causality	
Cardiovascular and muscular	MP-BR	FTA	
		Gain	GFAP (0.011)
		Causality	
	EMG → SBP	Causality	TNF-α (< 0.011), NfL (0.031)
Muscular	Muscles	Mean EMG	TNF-α (< 0.001), BDNF (0.030)
		Mean EMG impulse	TNF-α (< 0.001), BDNF (< 0.001)
		MVC of soleus	IL-6 (< 0.001), TNF-α (< 0.001), NfL (0.014), GFAP (0.015), BDNF (0.048)
		MVC of lateral gastrocnemius	
		MVC of medial gastrocnemius	t-Tau (0.045)
		EMG isometric contraction	
		EMG isotonic contraction	GFAP (< 0.001), NfL (< 0.001), UCH-L1 (0.022)
	Jump test	Peak velocity	IL-6 (< 0.001)
		Peak acceleration	
		Flight time	IL-6 (< 0.001)
		Flight height	IL-6 (0.031)
		Peak power	
	Sway	COPr	TNF-α (< 0.001), GFAP (0.011)
		COPrv	GFAP (0.004), NfL (0.011)

Significant correlations (robust false discovery rate [FDR] p < 0.05) between changes in physical/physiological variables and changes in serum biomarkers: Variable (robust FDR *p*-value). Data are presented for all participants. Inclusion criteria were set at FDR Huber M-estimation p < 0.05. Regardless of the change in physical or physiological variable, the biomarkers tracked in the opposite (red) or same direction (black) based on Spearman's coefficient (Figure 3). BP: blood pressure; FTA: fraction time active; HR → SBP: heart contractions changing systolic BP; MP-BR: muscle-pump baroreflex; EMG → SBP: leg muscle contractions changing systolic BP; MVC: maximum voluntary contraction; EMG: electromyography; COPr: average center of pressure sway radius; COPrv: average center of pressure sway velocity along the radius vector.

Changes in the biomarkers BDNF and TNF-α were predominantly associated with changes in heart rate, blood pressure, and muscle contractile force, while NfL and GFAP were mostly associated with baroreflex and motoneuron (EMG and posture) control. UCH-L1 was also significantly implicated in motoneuron activation during isotonic contraction. More interactions were seen for MVC of the soleus muscle than any other measure, with positive relationships with both pro-inflammatory (TNF-α, IL-6) and neural injury (NfL, GFAP, BDNF) markers.

Although most interactions were positively correlated (Spearman's), negative relationships were observed for TNF-α with systolic blood pressure and NfL with the causality of muscle contractions on blood pressure. Changes in the jump test results (peak velocity, flight time, and height) were negatively associated with IL-6.

Next, we investigated the quantitative relationship between sex (female, male) and intervention (control, exercise) using FDR with M-Huber estimation (*p* ≤ 0.05) (Table 4).

**Table 4:** Correlation across sex (female, male) and intervention (control, exercise) of individual changes in physical system markers from baseline (R0 to BDC-2) with the change in serum biomarkers (R1 to BDC4) using false discovery rate analysis with robust Huber M-estimation (*p* < 0.05).

			**Sex**		**Intervention**	
**System**	**Unit**	Δ **Physical/physiological variable**	**Females (*****n*** = **10)** Δ **biomarker**	**Males (*****n*** = **9)** Δ **biomarker**	**Control (*****n*** = **9)** Δ **biomarker**	**Exercise (*****n*** = **10)** Δ **biomarker**
Cardiovascular	Cardiac	HR	t-Tau (0.0005) IGF-1 (0.027)	TNF-α (0.017)	TNF-α (0.006)	
	Vascular	Systolic BP	BDNF (0.045)			
		Diastolic BP		IGF-1 (0.034) IL-6 (0.015) TNF-α (0.027) t-Tau (0.034)		
		Mean BP				
	Cardiac BR	FTA	UCH-L1 (< 0.0001)		IGF-1 (0.002)	NfL (0.001)
		Gain	IL-6 (0.027)		GFAP (0.039) UCH-L1 (0.045) IGF-1 (0.002)	
		Causality				
	HR → SBP	Causality				
Cardiovascular and muscular	MP-BR	FTA				
		Gain	Il-6 (0.0002) GFAP (0.032)	IL-6 (0.017) IGF-1 (0.001)		
		Causality			IL-6 (0.0001) UCH-L1 (< 0.0001)	
	EMG → SBP	Causality	NfL (0.001)		GFAP (0.033)	
Muscular	Muscles	MVC of soleus	GFAP (0.023)	t-Tau (0.002)		
		MVC of lateral gastrocnemius				
		MVC of medial gastrocnemius	t-Tau (< 0.0001) TNF-α (0.001) IGF-1 (0.045)	BDNF (0.016) GFAP (0.001)	t-Tau (< 0.0001)	IL-6 (0.024)
		EMG isometric contraction		t-Tau (0.004)		IL-6 (0.009)
		EMG isotonic contraction	GFAP (0.045)	TNF-α (0.039)		
	Jump test	Peak velocity		IGF-1 (0.001) IL-6 (0.009)		
		Flight time				
		Flight height		IGF-1 (0.029) IL-6 (0.007)		
		Peak power		t-Tau (0.016)		
	Sway	COPr		t-Tau (< 0.0001)		t-Tau (0.001)
		COPrv	t-Tau (0.028)	UCH-L1 (< 0.0001)	GFAP (0.015) NfL (< 0.0001)	t-Tau (0.012)

Significant correlations between changes in physical variables and changes in serum biomarkers: variable (false discovery rate, robust *p*-value). Data are presented for sex (combines the participants of a single sex from the control and exercise groups) and intervention (combines the participants of a single intervention from the female and male groups). Inclusion criteria were set at FDR Huber M-estimation *p* < 0.05. Regardless of the change in physical or physiological variable, the biomarkers tracked in the opposite (red) or same direction (black) based on Spearman's coefficient (Figure 3). BP, blood pressure; FTA, fraction time active; HR → SBP, heart contractions changing systolic BP; MP-BR, muscle-pump baroreflex; EMG → SBP, leg muscle contractions changing systolic BP; MVC, maximum voluntary contraction; EMG, electromyography; COPr, average center of pressure sway radius; COPrv, average center of pressure sway velocity along the radius vector.

In females, with respect to the cardiovascular system, standing heart rate was positively correlated with t-Tau, and vascular changes were positively correlated with IGF-1 and BDNF. Changes in baroreflex function were negatively related to changes in UCH-L1 and positively associated with IL-6 and GFAP. An increase in NfL was correlated with a decrease in muscle-pump coupling and changes in blood pressure. In relation to muscle contraction changes, GFAP was positively associated with MVC of the soleus and EMG generation during maximal isotonic contraction, while t-Tau, TNF-α, and IGF-1 were inversely associated with MVC of the medial gastrocnemius. Total tau was also directly correlated with sway velocity (COPrv).

In the male cardiovascular system, TNF-α was positively correlated with changes in HR, while IGF-1 was positively correlated and IL-6 was negatively correlated with blood pressure. Muscle activity was also related to biomarker changes. Total tau, BDNF, and GFAP changed in the opposite direction from changes in MVC. In addition, t-Tau was negatively correlated with EMG with maximal isometric contraction, and TNF-α changed directly with changes in EMG from maximal isotonic contractions. The metrics from the jump test were predominantly associated with the pro-inflammatory proteins IFG-1 and IL-6. Decreases in peak velocity, flight time, and flight height were correlated with biomarker increases. Changes in peak power were positively associated with t-Tau. Finally, increases in sway parameters were correlated with neurodegenerative markers. COPr increased with t-Tau, and COPrv increased with UCH-L1.

In the control group, 11 physiological measures were significantly correlated with one of the seven biomarkers, whereas in the exercise group, only five physiological measures were associated with three of the biomarkers. TNF-α was positively correlated with HR and COPr and negatively correlated with cardiac baroreflex gain. Inflammatory proteins, IGF-1 and IL-6, were positively correlated with HR and muscle-pump baroreflex FTA (Table 3). Similar to females, BDNF in the controls was positively correlated and solely associated with blood pressure. All correlations with UCH-L1 were positive and correlated with HR, contraction EMG, and postural sway. Only two significant correlations were observed in the exercise group: one with t-Tau, which was negatively correlated with diastolic blood pressure, and one with NfL, which had a positive correlation with cardiac baroreflex FTA.

### Discriminant analysis

Discriminant analysis for sex (Table 5), using the changes in biomarkers and physiological measures, revealed that the responses of t-Tau, COPr, muscle-pump baroreflex and gain, and MVC of the lateral gastrocnemius could identify female and male participants. With intervention (Table 6), discriminant analysis indicated that changes in three biomarkers (NfL, GFAP, and UCH-L1) and five physiological assessments (EMGimp, COPrv, muscle-pump baroreflex causality, MVC of the lateral gastrocnemius, and peak jump acceleration) could separate the control and exercise groups.

**Table 5:** Discriminant analysis of bed rest participants by sex (female, male).

	**Discriminants**				
	**t-Tau**	**COPr**	**Gain: MP-BR**	**Causality: MP-BR**	**MVC of LG**
*F*-ratio	25.0	10.1	7.0	2.1	18.0
Prob > *F*	0.0002	0.007	0.020	0.170	0.001
**Mean**					
Female (*n* = 10)	−0.12	5.00	0.10	−0.05	−0.15
Male (*n* = 9)	0.30	2.95	−0.37	−0.09	0.14
All (*n* = 19)^*^	0.08	4.03	−0.12	−0.07	−0.01

* Analysis used 19 participants (one male participant was missing muscle and jump data). Stepwise inclusion stopped when all were properly classified (entropy *r*^2^ = 0.99; −2log-likelihood = 0.192). Control participants were on 6° head-down tilt bed rest for 14 days without daily exercise, while the exercise group had 1 h daily of a combination of high-intensity interval training, aerobic, and strength exercises. MP-BR, muscle-pump baroreflex; MVC, maximum voluntary contraction; LG, lateral gastrocnemius. Biomarkers: t-Tau, total tau protein.

All physical/physiological measurements and biomarkers were made available for discriminant analysis. Variables entered the model in a forward stepwise manner by best probability until all participants were properly classified.

**Table 6:** Discriminant analysis of bed rest participants for interventions (control, exercise).

	**Discriminants**							
	**NfL**	**GFAP**	**UCH-L1**	**EMGimp**	**COPrv**	**Causality, MP-BR**	**MVC of LG**	**Jump peak acceleration**
*F*-ratio	8.4	2.1	15.3	8.5	31.8	9.5	0.05	2.7
Prob > *F*	0.016	0.178	0.003	0.016	0.0002	0.012	0.835	0.133
**Mean**								
Control (*n* = 9)	4.56	18.1	−1.81	−64.0	18.6	−0.094	0.121	−0.075
Exercise (*n* = 10)	6.49	21.3	2.48	−20.5	9.10	−0.047	−0.126	0.187
All (*n* = 19)^*^	5.58	19.8	0.448	−41.1	13.60	−0.069	−0.009	0.063

All physical/physiological measurements and biomarkers were made available for discriminant analysis. Variables entered the model in a forward stepwise manner by best probability until all participants were properly classified.

* Analysis used 19 participants (one male control participant was missing muscle and jump data). Stepwise inclusion stopped when all were properly classified (entropy *r*^2^ = 0.99; −2log-likelihood = 0.246). Control participants were on 6° head-down tilt bed rest for 14 days without daily exercise, while the exercise group had 1 h daily of a combination of high-intensity interval training, aerobic, and strength exercises. MP-BR, muscle-pump baroreflex; MVC, maximum voluntary contraction; LG, lateral gastrocnemius; EMGimp, electromyogram impulse—the area of the rectified EMG signal between heartbeat R wave peaks (RR interval); COPrv, average center of pressure sway velocity. Biomarkers: NfL, neurofilament light chain; GFAP, glial fibrillary acidic protein; UCH-L1, ubiquitin carboxy-terminal hydrolase L1.

When applied to the four distinct groups (Table 7), a combination of eight variables was required: two biomarkers (t-Tau, IGF-1), three related to the muscle-pump baroreflex (gain, causality, EMGimp), two associated with muscle strength (MVC LG, peak jump power), and one with posture (COPrv).

**Table 7:** Discriminant analysis of bed rest participants for all conditions (female controls, female exercise, male controls, male exercise).

	**Discriminants**							
	**t-Tau**	**IGF-1**	**Gain MP-BR**	**Causality MP-BR**	**EMGimp**	**MVC of LG**	**Peak jump power**	**COPrv**
*F*-ratio	51.7	3.83	9.63	11.0	14.8	34.4	28.7	13.0
Prob > *F*	< 0.0001	0.057	0.005	0.003	0.001	< 0.0001	< 0.0001	0.002
**Mean**								
Female control (*n* = 4)	−0.158	−116	0.054	−0.073	−24.2	0.00043	0.475	27.2
Female exercise (*n* = 6)	−0.093	−155	0.132	−0.031	−24.0	−0.244	−0.688	9.81
Male control (*n* = 5)	0.048	−28.4	−0.265	−0.111	−95.8	0.217	−0.754	11.7
Male exercise (*n* = 4)	0.615	26.0	−0.499	−0.071	−15.1	0.052	−0.049	8.05
All (*n* = 19)^*^	0.080	−75.2	−0.122	−0.069	−41.1	−0.009	−0.326	13.6

All physical/physiological measurements and biomarkers were made available for discriminant analysis. Variables were entered into the model in a forward stepwise manner by best probability until all participants were properly classified.

* Analysis used 19 participants (one male control participant was missing muscle and jump data). Stepwise inclusion stopped when all were properly classified (entropy *r*^2^ = 0.99; −2log-likelihood = 0.192). Control participants were on 6° head-down tilt bed rest for 14 days without daily exercise, while the exercise group had 1 h daily of a combination of high-intensity interval training, aerobic, and strength exercises. MP-BR, muscle-pump baroreflex; MVC, maximum voluntary contraction; LG, lateral gastrocnemius; EMGimp, electromyogram impulse—the area of the rectified EMG signal between heartbeat R wave peaks (RR interval); COPrv, average center of pressure sway velocity. Biomarkers: t-Tau, total tau protein; IGF-1, insulin-like growth factor 1.

### Interactions with baroreflex function

**Figure 5:**
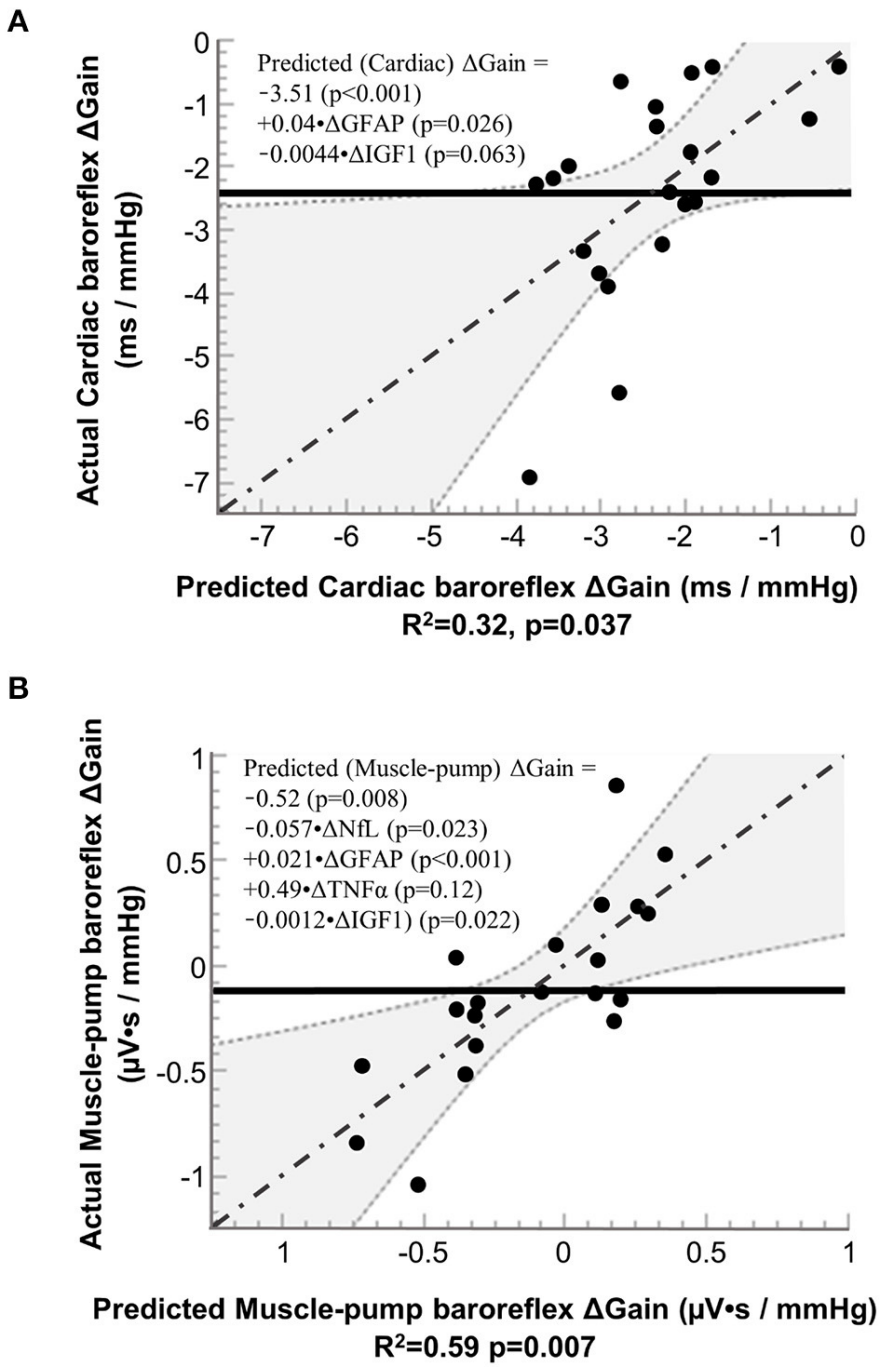
Results from stepwise regression analysis of pre-to-post (R+0 – BDC-2) changes (Δ) in neural injury biomarkers with pre-to-post (R+0 – BDC-1) changes in the gain of **(A)** cardiac baroreflex and **(B)** muscle-pump baroreflex. NfL, neurofilament light chain; GFAP, glial fibrillary acidic protein; TNF-α, tumor necrosis factor alpha; and IGF-1, insulin-like growth factor 1. Solid line (^**−−−−−−−**^): mean value of actual; dashed lines and shaded areas (……): 95% confidence area; dash dotted line (-·-·-·-·): regression line.

Based on inclusion into the multiple regression model for cardiac baroreflex, IGF-1 had a negative association, and GFAP had a positive association (Figure 5A). We also observed similar results for IGF-1 and GFAP with muscle-pump baroreflex, with an additional negative association for NfL and a positive association for TNF-α (Figure 5B).

## Discussion

In this first-of-its-kind study at the intersection of spaceflight with aging and inactivity, we show that in older persons, (i) 14 days of simulated spaceflight using 6° head-down tilt bed rest promotes signs of neurodegenerative processes independent of 60- to 75-min daily rigorous exercise, and (ii) many of these biomarkers correlate with observed changes in cardiovascular and muscular function.

### Elevated neural injury and inflammatory biomarkers

The global increase in neural injury markers and inflammatory cytokines in our participants is a powerful indicator of the severity of imposed inactivity in the 6° head-down position. This environment not only results in the physical deconditioning of these participants (Sadeghian et al., 2022) but is also associated with neurological insult. Since these changes occurred independent of the specific space-based exercise intervention, these data suggest that the 1 h daily of applied exercise did not overcome the 23 h of inactivity. As these data were collected in the head-down and not supine position, the application to hospital bed rest is not direct, but the rapidity with which these changes occurred indicates that they are important to monitor during extended immobilization as indicators of post-intensive care syndrome (Voiriot et al., 2022).

#### Neural injury (NfL, GFAP)

The increase in NfL levels from the beginning to the end of the bed rest reflects similar changes reported from cosmonauts (Zu Eulenburg et al., 2021). They reported an average NfL level increase of 33% (11.4–15.2 pg/mL) in five male cosmonauts [49.2 (2.7) (SEM) years], which closely mimics the changes observed in our bed rest participants. However, the mean flight duration of the astronauts on the international space station was 169 days, 12 times longer than our older participants spent in HDBR. Furthermore, the effect of the spaceflight and its analog HDBR on overall NfL levels could be a cause for concern. Rising serum NfL levels with reduced activity have been associated with cognitive decline (Desai et al., 2022) and all-cause mortality in older adults (Nguyen et al., 2022).

Considering that rising NfL levels signal the early onset of neural injury as well as many neurological complications (Ferreira-Atuesta et al., 2021; Verde et al., 2023), these data indicate that the HDBR is an effective model for weightlessness exposure in relation to neurological insults to the brain. We hypothesized that the headward fluid shifts observed in HDBR would predispose participants to this type of injury. The elevations in NfL we observed in older adults after 2 weeks of HDBR and those observed in five cosmonauts (Zu Eulenburg et al., 2021) are not unlike the reported changes in NfL from athletes with concussions (Shahim et al., 2017). One consideration for investigation should be the relationship between the headward fluid shift experienced in both HDBR and spaceflight by test participants and the brain, which may be a physical link between microgravity and concussion.

#### GFAP

The results from five male cosmonauts showed a 20% increase in GFAP levels (169–215 pg/mL) (Zu Eulenburg et al., 2021), which were similar in magnitude to the HDBR participants. The elevation in GFAP in our older adults in HDBR is notable in that it not only confirms, in a spaceflight analog, the cosmonaut results, but that similar increases are emerging as a useful prognostic and diagnostic marker for multiple neurological diseases (Abdelhak et al., 2022). Prior to the latest human spaceflight results (Zu Eulenburg et al., 2021), investigations of GFAP levels in altered gravity had shown varying results. After 14 days of microgravity exposure, the expression of astrocyte-specific GFAP was observed to decrease significantly (Day et al., 1998); however, the same effect was not observed with long-term microgravity exposure (Bellone et al., 2016). In an animal study (Grigoryan et al., 2012), GFAP levels increased during exposure to long-term microgravity. Mao et al. reported an increase in perivascular reactive GFAP astrocytes in the hippocampus of mice who had spent 35 days on the International Space Station when compared to control mice on the ground (Mao et al., 2020). Future research should examine the correlation of GFAP levels with long-term human spaceflight and physical inactivity and determine the directionality of such correlation.

#### Growth factors (IGF-1)

In a study of four male and four female astronauts, Hughson et al. (2016) reported elevated IGF-1 following 6 months on the International Space Station (ISS). However, they indicated that female participants tended to have smaller changes. In the current study, we found a reduction in IGF-1 in the female participants, with no change in the males. The results from the analog and space environments suggest that sex differences reduce the gravitational influence on IGF-1. Bed rest is analogous and does not completely match the spaceflight environment. Although the exercises were space-based, they were not the same, and the duration of bed rest was ~10% of that of the average crewmember's flight.

Previous animal models in microgravity research had suggested that a suppression in the production of IGF-1 is most common when bones are not exposed to mechanical loading (Kumei et al., 2002). In particular, Kumei et al. (2002) observed reduced mRNA levels for IGF-1 in rat osteoblast cultures during spaceflight compared to ground controls. They also found that microgravity completely suppressed the expression of insulin receptor substrate-1, a molecule involved in IGF-1 signaling. The results from our study may suggest that HDBR inactivity in older adults promotes physical deconditioning (Aagaard et al., 2010; Jasim et al., 2017), and the reduction in IGF-1 in the female participants suggests a greater or earlier susceptibility of older females to bed rest.

#### Pro-inflammatory cytokines (TNF-α and IL-6)

We observed significant increases in TNF-α and IL-6 following HDBR. Previous studies investigating the levels of TNF-α and IL-6 during bed rest have indicated an increase in overall levels of these cytokines. Notably, bed rest is often associated with bone and muscle mass loss and is an overall stressor on the cardiovascular system. Previous investigations have also shown that both young and older populations have an increase in overall TNF-α and IL-6 levels. However, the older population showcases even more pronounced levels of TNF-α and IL-6, which can potentially signal an increase in inflammatory markers with immobility and increasing age.

Although the results of serum and urinary data for cytokines in astronauts are mixed, there is evidence of their elevation during space missions (Thiel et al., 2017). A study of endothelial cell cultures has shown a marked increase in IL-6 production up to 3 months post-flight (Muid et al., 2013).

In a study of mice exposed to 35 days of microgravity, Mao et al. (2020) described a relationship between pro-inflammatory cytokines and damage to the blood–brain barrier (BBB) and related pathways to spaceflight. The study found evidence of elevated proteins associated with apoptosis and a decrease in tight junction proteins. The combination of decreased tight junction proteins, increased GFAP, and the presence of apoptosis markers could have led to damage to the BBB. Mao et al. also found that the Cdk5 signaling pathway, which is essential for the cellular mechanisms behind cognition and regulation of neural cell death and is associated with the NfL, Tau, and BDNF pathways (Pao and Tsai, 2021), showed remarkable downregulation after exposure to spaceflight (Mao et al., 2020).

With the similarity between HDBR and data collected from human and animal models in space, we recommend integrating measures of neural function with biomarkers of neural injury into mission planning and as a component of exercise prescription. The parallels in neurodegenerative markers and inflammatory proteins with the animal models could indicate that the cerebral impact of the head-down position may not only mimic fluid shifts in spaceflight but may also provoke BBB damage. Further research into the Cdk5 pathway in HDBR and spaceflight is recommended.

### Serum markers and physiological measurements

Four of eight serum markers showed consistent change with bed rest independent of sex and intervention, while a fifth changed in females but not males and independent of intervention. To further investigate the significance of all eight serum markers, we examined their relationship with changes in cardiovascular and muscular system physiology. Did physiological function change over bed rest in a manner consistent with the change (positive or negative) in a serum marker? With intra-individual variability associated with differences in participant physiology, it was expected that responses to bed rest may not have been similar in magnitude or direction and that this would mask biomarker–physiological function relationships when investigated as group effects. However, if the underlying physiological processes were similar, then any changes observed in physiological function would be reflected in consistent changes in their associated biomarkers and could be tested with correlation analyses.

Spearman's ranked test was first applied to find the directionality and strength of correlation. Given the large number of multiple comparisons, false discovery rate analysis (FDR) was used. The robust Huber M-estimation was used due to non-normally distributed data and outlier effects. Because of the complexity of conducting a bed rest project involving older people, only a small number of participants in each subgroup was feasible. Therefore, we have limited in-depth discussion of the effects associated with bed rest, with links to possible sex or intervention differences. Finally, Spearman's rank test, FDR, discriminant, and multiple regression analyses are inferential, not causal, and are used to identify areas of interest for future targeted research.

#### Heart rate and blood pressure

Two serum markers, namely TNF-α and BDNF, were associated with the cardiovascular system when all participants were grouped together. Changes in TNF-α were correlated with elevated cardiovascular stress during standing, while BDNF may have had a repairing role in the presence of cardiovascular stressors.

TNF-α is secreted by several cardiovascular cells (endothelial, smooth muscle, and cardiac muscle) and has wide-ranging effects on these systems through cardiac muscle function and vasodilation (Urschel and Cicha, 2015). The involvement of TNF-α with both HR and blood pressure is therefore consistent with its sites of secretion and action. Increases in TNF-α were correlated with decreases in blood pressure and increases in HR. The behavior of TNF-α was consistent with this hypothesis as HR and blood pressure are inversely related through the baroreflex; decreases in blood pressure led to increases in HR. Those participants with the greatest increase in orthostatic challenge (i.e., largest drop in blood pressure upon standing) had the largest increase in TNF-α.

Although we did not observe a significant change in BDNF with bed rest, we detected a positive correlation between BDNF levels and blood pressure. This would suggest that while the BDNF response to bed rest was variable between participants, it changed in the same direction with a similar relative order of magnitude as blood pressure in these participants.

BDNF is secreted by the central nervous system, lungs, heart, and liver and plays a key role in protecting neurons in both the central and peripheral nervous systems. Responses to orthostatic stress are most characterized by alterations in HR via vagal withdrawal and increased involvement of vasoconstrictor tone (Blaber et al., 2022). Previous research has shown that a decrease in overall BDNF levels has been associated with neurodegenerative disorders such as Alzheimer's and Parkinson's disease (Phillips et al., 1991; Levivier et al., 1995; Connor et al., 1997; Parain et al., 1999; Howells et al., 2000; Michalski and Fahnestock, 2003). Further research has shown that when exposed to acute stressors, after an initial decrease, an increase in BDNF levels is observed. Such a pattern was observed with 14 days of bed rest (Soavi et al., 2016). The increase in BDNF levels was thought to act as a restorative to combat the stressor and was shown to partially repair the metabolic damages caused by inactivity (Soavi et al., 2016).

The current study is consistent with the hypothesis that BDNF has a repairing role. The positive correlation with blood pressure levels could suggest the factor's repair effect to combat the physiological downsides caused by 14 days of head-down bed rest, especially to the blood pressure regulating mechanisms. For example, participants with larger secretions of BDNF would be associated with smaller decreases or even increases in BP upon standing. Participants with lower or reduced BDNF, and therefore less protection, would be more susceptible to blood pressure declines with standing.

#### Cardiac and muscle-pump baroreflex

The results from this study show an interaction between the individual cytokines and neural injury biomarkers with cardiac and muscle-pump baroreflexes, which could provide insight into the potential role of inflammatory responses and neurodegeneration in spaceflight and aging. Changes in NfL and GFAP were significantly correlated with baroreflex function, while NfL, GFAP, IGF-1, and TNF-α emerged as multiple regression coefficients for baroreflex gain.

In our previous analyses of cardiovascular control in the same participants, we showed a degradation in the neural and not the mechanical components of the cardiac and muscle-pump blood pressure feedback loop, which was not improved with exercise (Sadeghian et al., 2022). We reported similar cardiac muscle-pump baroreflex impairments with long-term (60 days) bed rest (Xu et al., 2020) and cardiac baroreflex with short-duration (8–16 days) spaceflight (Blaber et al., 2022). We proposed that these could result from both inactivity and cephalad fluid shifts associated with HDBR and weightlessness.

Correlation analysis indicated a significant positive relationship between NfL and cardiac baroreflex FTA, which were significantly reduced after bed rest. We also observed a significant increase in NfL with bed rest. The production of NfL occurs within the cell bodies of neurons located in the central nervous system (CNS) and peripheral nervous system (PNS), and its production is triggered by neuronal damage or dysfunction (Yuan et al., 2012; Khalil et al., 2018). While the specific triggers for NfL production are not fully understood, it is known that various pathological processes affecting neurons, such as neurodegenerative diseases (Rosengren et al., 1996; Gaiottino et al., 2013; Khalil et al., 2018; Gaetani et al., 2019), traumatic brain injury (Snowdon et al., 1997; Thelin et al., 2017), or neuronal damage (Strydom et al., 2018), can lead to increased production and the release of NfL into the surrounding tissues and blood. Elevated levels of NfL in the blood are indicative of neuronal injury or degeneration, as the protein leaks out from damaged neurons (Gaetani et al., 2019). In addition to the CNS, peripheral neuropathies and injuries affecting peripheral nerves can also result in elevated NfL levels (Strydom et al., 2018).

GFAP had a positive coefficient with muscle-pump baroreflex gain. Like NfL, the production of GFAP occurs predominantly in the brain and spinal cord (Huang et al., 2019). Astrocytes are the main source of GFAP synthesis (Rodnight et al., 1997); however, GFAP expression is not exclusive to astrocytes, as it can also be found in other glial cell types (Eng et al., 2000). Triggers that upregulate the expression of GFAP (Rodnight et al., 1997) include brain injury (Thelin et al., 2017), neuroinflammation, ischemia, infection, neurodegenerative diseases (Pelinka et al., 2004), and other insults that lead to astrocyte activation or reactive gliosis.

Elevated levels of GFAP can be detected in cerebral spinal fluid (CSF) and blood following CNS injuries or neurodegenerative conditions (Middeldorp and Hol, 2011), indicating astrocyte reactivity and the extent of CNS damage. GFAP expression and reactivity in astrocytes can be both beneficial and detrimental, depending on the context (Escartin et al., 2021). In this study, we observed a global increase in GFAP along with a positive correlation with muscle-pump baroreflex gain. This could indicate a possible neurological injury associated with skeletal muscle activation in the blood pressure control system, for which GFAP may have been beneficial since a higher astrocyte response was associated with better baroreflex gain than a lower response.

To further explore the interaction of inflammation, neural injury, and blood pressure regulation, we performed multiple regression analysis of the serum biomarker data with cardiac and muscle-pump baroreflex gain.

Multiple regression analysis for cardiac baroreflex gain showed a positive relationship with GFAP and a negative relationship with IGF-1, variables not seen with FDR. However, the assumptions for the two statistical procedures are different. The former looks at the probability of correlation between paired variables over multiple comparisons, and the latter determines the relative contribution of a specific combination of variables to predicting a change in a single variable. These results indicate a possible relationship between neurodegenerative effects on cardiac baroreflex function and the observed increase in GFAP, similar to the FDR analysis of muscle-pump baroreflex. The negative IGF-1 relationship parallels aging effects (Aagaard et al., 2010; Jasim et al., 2017).

Multiple regression analysis of the muscle-pump baroreflex indicated the additional involvement of NfL and TNF-α compared to the cardiac baroreflex. This may reflect additional complexity, as the muscle-pump baroreflex involves both autonomic and motoneuron recruitment. The positive coefficient for TNF-α on baroreflex gain is consistent with its relationship with the cardiovascular system observed in the correlational analysis, where it was related to increased HR and reduced blood pressure during standing. The negative coefficient for NfL with muscle-pump gain is consistent with the hypothesis of neurological insult, resulting in a reduced reflex response to blood pressure.

Although our previous results indicated no effect of bed rest on the causal relationship between muscle activity and blood pressure (EMG → SBP, muscle-pump mechanics) (Sadeghian et al., 2022), a correlational analysis of NfL showed a significant negative correlation. These data suggest neural degradation, which impacts afferent and efferent components of the reflex, and may explain the extended time for muscle-pump reflex deficits following bed rest when compared to the cardiac baroreflex (Xu et al., 2020).

#### Muscle contraction, postural sway, and jump test

An examination of tests of postural sway and muscle strength, control, and power revealed significant correlations with neurodegenerative and pro-inflammatory markers following HDBR. Together, these biomarkers could be leading indicators for the onset of degenerative processes in the skeletal muscle pathways related to reduced motor unit recruitment and/or remodeling during inactivity.

Like the cardiovascular system, changes in TNF-α, BDNF, and GFAP were correlated with a wide variety of muscular variables. TNF-α was positively correlated with changes in muscle EMG, soleus MVC, and COPr. GFAP was associated with soleus MVC, isotonic contraction EMG, and postural sway (COPr, COPrv). BDNF was significantly correlated with EMG during standing and soleus MVC. paired with in relation to We also observed significant decreases in these same physical and physiological responses with bed rest. This may indicate a strong influence of inflammation (TNF-α) and the recruitment of neural restorative measures (BDNF), as well as MVC of the soleus.

The neural injury marker, t-Tau, correlated negatively with the MVC of the medial gastrocnemius. Tau is a protein that stabilizes the structure of neurons in the brain. Total tau is a biomarker that measures the total amount of tau protein in the blood or cerebrospinal fluid. Although there is limited prior research on how t-Tau levels may change after bed rest, studies have shown that t-Tau increases with age and may be a marker of age-related cognitive decline and neurodegeneration (Harrison et al., 2019; Huseby et al., 2019). Regarding neurodegenerative diseases, t-Tau levels tend to be elevated in the cerebrospinal fluid of affected individuals (Harrison et al., 2019). This increase is believed to be due to the release of tau protein from degenerating neurons in the brain (Huseby et al., 2019).

We found that UCH-L1 was positively correlated with the change in isotonic contraction. Ubiquitin C-terminal hydrolase L1 (UCH-L1) is a protein that is primarily expressed in neurons and plays an important role in development. UCH-L1 has been identified as a potential biomarker for traumatic brain injury and other neurological conditions (Yang et al., 2023). There is limited research on how UCH-L1 levels may change after bed rest or with aging specifically. However, studies have shown that UCH-L1 levels can be affected by a variety of factors, including traumatic brain injury, neurodegenerative diseases, and aging (Reinicke et al., 2019). In the case of traumatic brain injury, UCH-L1 levels may increase due to neuronal damage. Similarly, in neurodegenerative diseases, UCH-L1 levels may increase as a result of neuronal damage and loss (Vinciguerra, 2019). UCH-L1 levels tend to decrease with age (Reinicke et al., 2019), possibly due to a decline in the activity of the ubiquitin-proteasome system in neurons. This decline in UCH-L1 activity may contribute to age-related cognitive decline and an increased risk of neurological disorders (Yang et al., 2023).

Interleukin-6 had its primary interaction with the jump test. It was significantly and negatively correlated with peak velocity, flight time, and flight height, which all had significant reductions after bed rest while also being positively correlated with soleus MVC. Immune cells (Ferguson-Smith et al., 1988) are the primary source of IL-6; however, IL-6 can also be secreted by endothelial cells, fibroblasts, and certain tumor cells (Ferguson-Smith et al., 1988). The effects of IL-6 are diverse and context-dependent, involving the regulation of inflammation, hematopoiesis (the production of blood cells), and the acute-phase stress response. Additionally, it impacts multiple organ systems, such as the immune system, cardiovascular system (Kanda and Takahashi, 2004), nervous system (Zhou et al., 2021), musculoskeletal system (Muñoz-Cánoves et al., 2013), and the liver (Hsieh et al., 2019). Bed rest, in general, can lead to muscle wasting and atrophy, resulting in a decrease in the production of anti-inflammatory cytokines (Drummond et al., 2013). This can cause an imbalance in the inflammatory response, potentially leading to increased levels of pro-inflammatory cytokines such as IL-6 (Drummond et al., 2013).

IL-6 plays a significant role in regulating muscle power and strength (Park et al., 2013) and is crucial for the body's inflammatory and immune response to stress (Ferguson-Smith et al., 1988). Some studies indicate that moderate increases in IL-6 can enhance muscle force production and overall performance (Ferrucci et al., 2002; Pereira et al., 2009; Park et al., 2013), likely due to its ability to facilitate glucose uptake, providing more energy to the working muscles (Park et al., 2013) (measured in this study by the MVC test). This may explain the positive correlation between IL-6 and the soleus MVC test. On the other hand, maintaining a balance in IL-6 levels is essential because chronic elevation caused by inflammation can have adverse effects on muscle power and function (Haddad et al., 2005). In the current study, IL-6 had the most interactions with the jump test, which is a measure of muscle power.

### Sex and exercise

In our previous publication (Sadeghian et al., 2022), we found that the reduction in baroreflex was not affected by exercise. Following bed rest, similar numbers of females and males presented with presyncopal symptoms during the 5-min stand (Sadeghian et al., 2022) and 15-min tilt test (Hajj-Boutros et al., 2023). Given the small number of participants, we therefore examined the effects of sex by combining control and exercise participants within sex and intervention by combining male and female participants within intervention. The biomarker correlations displayed different patterns when examined by sex, although most biomarkers were found to be significantly related to a physical/physiological variable in either one. The males had a greater number of negative interactions compared to the females, with the majority being inflammatory proteins (IL-6, TNF-α, and IGF-1) in blood pressure, muscle-pump baroreflex, and jump test results. This may indicate a greater inflammatory response to HDBR in males compared to females. In our article on baroreflex function in these participants (Sadeghian et al., 2022), we found a greater reduction in muscle activation and muscle-pump control during standing in the males. This is evident in the data presented here, where male participants had twice the reductions in muscle-pump baroreflex indices compared to females.

When we compared control with exercise participants, we observed a reduction in the number of significant correlations. Both control and exercise groups had fewer correlations than either the female or male groupings. In the control group, the inflammatory marker TNF-α was associated with cardiac control, while IGF-1 and IL-6 were associated with the muscle-pump baroreflex. The neurodegenerative markers GFAP, UCH-L1, and NfL were correlated with reflex mechanisms: cardiac and muscle-pump baroreflexes, and postural sway. The exercise group, on the other hand, only had minimal neurodegenerative biomarker correlations: NfL with cardiac baroreflex and t-Tau with the jump test and postural sway. These data support the hypothesis that the exercise program would reduce the effects of bed rest on neural injury. However, the correlation of NfL with cardiac baroreflex and sway, along with the lack of protection against orthostatic intolerance (Sadeghian et al., 2022), are strong indicators that these exercises were not effective for maintaining the cardio-postural control system (Xu et al., 2017, 2020).

Several serum and physical/physiological markers were identified through discriminant analysis. These could provide further information on the whole-body impact of bed rest in relation to sex and exercise intervention in older persons. Unlike correlational analysis, where we obtained data on links between biomarkers and physical outcomes, discriminant analysis provides an avenue to investigate distinguishing characteristics related to specific groups. With main effects and all subgroup comparisons, cardiac-specific variables were not returned as discriminatory measures. This may indicate that their responses were similar across all groups. Indeed, we previously reported that these participants' HR and cardiac baroreflex responses were universally impacted (Sadeghian et al., 2022) by bed rest.

Instead, serum markers and muscle-related measures were highlighted, with each containing at least one biomarker, one muscle-pump variable, and a muscle strength measurement. These data show that the male and female participants responded differently to bed rest in combination with the intervention. These differences were related to a greater negative effect of t-Tau and IGF-1 and the combined effects of motor control associated with muscle-pump baroreflex, calf muscle contractions, jumping power, and postural sway.

### Strengths and limitations

Through quantitative serum analysis, we showed significant increases in inflammatory and neurodegenerative biomarkers over a 14-day exposure to HDBR in otherwise healthy 55- to 65-year-olds. Our data are consistent with recently reported data from cosmonauts, and we have provided additional markers to be considered. The exercise intervention had no significant impact on these changes, nor did it prevent a significant loss of orthostatic tolerance. Because of the logistical complexities of mounting a study like this, only a few male or female participants (4–6) were in each category. This limited our ability to investigate the sex/intervention interactions for the biomarkers from the ANOVA analysis; however, we have presented the means (95% CI) as pilot data for future comparisons.

For the first time, we had the opportunity, as part of a dedicated head-down tilt bed rest study with older persons, to investigate cardio-postural control and biomarkers of neural injury in males and females with an exercise intervention. This provided unique data in relation to post-HDBR and possible insights into spaceflight neural deconditioning. We examined the correlation between the changes in these biomarkers with a robust false discovery rate analysis correcting for multiple comparisons. This revealed relationships between neurodegenerative biomarkers and blood pressure and posture control, providing evidence in support of cardio-postural impairment through HDBR-associated neural injury. The association of neural injury (GFAP, NfL), growth factors (IGF-1), and pro-inflammatory (TNF-α) markers was also supported through multiple regression analysis. Both correlational and multiple regressions do not indicate causal relationships and are only used here to provide support to hypothesize and form the base for future studies.

As with the analysis of biomarkers by ANOVA, we were limited in our ability to test relationships between biomarkers and physiological measurements at the sex/intervention level. However, the use of discriminant analysis, which used the full data set, provided important information regarding the influence of serum markers and physiological outcomes in relation to both sex and exercise and their respective combinations.

This study was one of eight research groups embedded in the HDBR project. Our hypotheses focused on cardio-postural control declines with bed rest, making many measurements beyond the scope of our study. For example, we were unable to relate the changes observed with the neurodegenerative biomarkers to associated cognitive and psychological outcomes. Furthermore, the multi-modal exercise intervention was designed by an external expert committee (Hedge et al., 2022), within whose framework all funded research programs functioned. As such, it is difficult to relate current observations to specific exercises. Analyses exploring these aspects will be pursued in future publications from us and the other teams participating in the first Canadian aging and inactivity study.

## Conclusion

These data suggest that even a short period (9–14 days) of HDBR in older persons can elevate major protein markers of neural injury, which were correlated with changes in the cardiovascular and muscular systems, and in particular those systems involving the baroreflex and postural sway. Additionally, discriminant and multiple regression analyses point to neurodegenerative components associated with baroreflex impairment following HDBR that exercise was unable to halt. This raises significant concerns regarding post-bed rest recovery and its long-lasting effects, particularly in the elderly. Finally, these results highlight the need for neurological monitoring of astronauts as space missions become longer.

## Data availability statement

The datasets presented in this article are not readily available because data may only be shared for the use under which it was ethically approved. Requests to access the datasets should be directed to andrew_blaber@sfu.ca.

## Ethics statement

The studies involving humans were approved by McGill University Health Centre Research Ethics Board, Office of Research Ethics at Simon Fraser University. The studies were conducted in accordance with the local legislation and institutional requirements. The participants provided their written informed consent to participate in this study.

## Author contributions

AB had full access to all the data in the study and took responsibility for the integrity of the data and the accuracy of the data analysis. AB and IS contributed to the concept and design and after obtaining funding, AB coordinated and supervised data acquisition and statistical analysis. FS and DN conducted all sit-to-stand tests and collected and analyzed muscle activity, jump test, and baroreflex function data. All contributed to interpreting the data and drafting the manuscript. All authors contributed to the article and approved the submitted version.
